# Polymer Identification and Specific Analysis (PISA) of Microplastic Total Mass in Sediments of the Protected Marine Area of the Meloria Shoals

**DOI:** 10.3390/polym13050796

**Published:** 2021-03-05

**Authors:** Valter Castelvetro, Andrea Corti, Jacopo La Nasa, Francesca Modugno, Alessio Ceccarini, Stefania Giannarelli, Virginia Vinciguerra, Monica Bertoldo

**Affiliations:** 1 Department of Chemistry and Industrial Chemistry, University of Pisa, 56124 Pisa, Italy; andrea.corti@unipi.it (A.C.); jacopo.lanasa@for.unipi.it (J.L.N.); francesca.modugno@unipi.it (F.M.); alessio.ceccarini@unipi.it (A.C.); stefania.giannarelli@unipi.it (S.G.); virginia.vinciguerra@dcci.unipi.it (V.V.); 2 CISUP—Center for the Integration of Scientific Instruments of the University of Pisa, University of Pisa, 56124 Pisa, Italy; 3 Department of Chemical, Pharmaceutical and Agricultural Sciences, University of Ferrara, via L. Borsari, 45121 Ferrara, Italy; brtmnc@unife.it; 4 Institute of Organic Synthesis and Photoreactivity, National Research Council of Italy (ISOF-CNR), via P. Gobetti 101, 40129 Bologna, Italy

**Keywords:** microplastics, marine sediment, pet, nylon 6, nylon 6,6, reversed-phase HPLC, polyolefin, polystyrene, Pyr-GC/MS, polymer degradation

## Abstract

Microplastics (MPs) quantification in benthic marine sediments is typically performed by time-consuming and moderately accurate mechanical separation and microscopy detection. In this paper, we describe the results of our innovative Polymer Identification and Specific Analysis (PISA) of microplastic total mass, previously tested on either less complex sandy beach sediment or less demanding (because of the high MPs content) wastewater treatment plant sludges, applied to the analysis of benthic sediments from a sublittoral area north-west of Leghorn (Tuscany, Italy). Samples were collected from two shallow sites characterized by coarse debris in a mixed seabed of *Posidonia oceanica*, and by a very fine silty-organogenic sediment, respectively. After sieving at <2 mm the sediment was sequentially extracted with selective organic solvents and the two polymer classes polystyrene (PS) and polyolefins (PE and PP) were quantified by pyrolysis-gas chromatography-mass spectrometry (Pyr-GC/MS). A contamination in the 8–65 ppm range by PS could be accurately detected. Acid hydrolysis on the extracted residue to achieve total depolymerization of all natural and synthetic polyamides, tagging of all aminated species in the hydrolysate with a fluorophore, and reversed-phase high performance liquid chromatography (HPLC) (RP-HPLC) analysis, allowed the quantification within the 137–1523 ppm range of the individual mass of contaminating nylon 6 and nylon 6,6, based on the detected amounts of the respective monomeric amines 6-aminohexanoic acid (AHA) and hexamethylenediamine (HMDA). Finally, alkaline hydrolysis of the residue from acid hydrolysis followed by RP-HPLC analysis of the purified hydrolysate showed contamination by polyethylene terephthalate (PET) in the 12.1–2.7 ppm range, based on the content of its comonomer, terephthalic acid.

## 1. Introduction

Plastic microparticles, commonly referred to as microplastics (MPs), either deriving from the environmental degradation of larger plastic waste items [1,2,3] or directly released as primary microparticles (microbeads, textile microfibers) in wastewaters, are a class of pollutants detected in virtually all natural environments, from oceans to inland waters [4], soils and even as airborne material [5], reaching such remote areas as the Arctic and Antarctica [6,7]. The ubiquitous presence of MPs, and likely so also of their ultimate products of further degradation into sub-micrometer sized particles (nanoplastics) [8], along with incipient evidence of their adverse interaction with living organisms [9], has stimulated increasing research efforts aimed at understanding their transport, distribution and fate [10,11,12]. Due to their small size microplastic can be ingested by various organisms at all trophic levels, and increasing scientific evidence highlights the possibility of their transfer into animal tissue and up the food chain reaching humans [13,14].

The most common synthetic polymers in plastic waste are polyolefins (polypropylene, PP, high density polyethylene, HDPE, and low-density polyethylene, LDPE) and polystyrene (PS), widely used in packaging and single-use disposable items such as tableware; polyester (mainly polyethylene terephthalate, PET, used for beverage bottles, packaging and as staple textile fiber) and polyamides (often referred to according to the tradename nylons) represent an additional significant fraction of MPs pollution. In the case of polyolefins, the environmental degradation processes are mainly ascribed to photo-oxidation, resulting in oxygen pickup due to free radical reactions with cascade effects eventually leading to polymer chain fragmentation and insertion of oxidized functional groups (carbonyls, carboxyl, hydroxyl, etc.) [15]. Such chemical transformations bear several consequences: (i) the initially high molecular weight is reduced and the polymeric material becomes more brittle, promoting progressive fragmentation into increasingly smaller particles; (ii) its density and hydrophilicity increase along with surface polarity and reactivity, enhancing adsorption/absorption of low molecular weight organic (including toxic polycyclic aromatic hydrocarbons, PAHs, and polychlorinated biphenyls, PCBs) and inorganic (heavy metals) environmental pollutants; (iii) increased wettability and specific surface area facilitate biofouling and adhesion inorganic particulate, all of the above promoting sinking down the water column and deposition in both shore and benthic sediments [16,17,18,19]. It has been estimated that less than 1% of the 5–12 million tons per year of plastics entering the oceans stays afloat for a long time, the remaining fraction reaching the seabed either in a very short time (this is the case of larger items of higher density plastics such as e.g., PET or low-density polymers with inorganic fillers) or over longer periods regardless of the initial density because of the abovementioned degradation and fouling phenomena [12,20,21].

Here we report the results of the application of our recently developed analytical protocol to the quantitative determination of the total mass content of a well-defined set of microplastics [22], hereafter Polymer Identification and Specific Analysis (PISA), in benthic marine sediments. The PISA protocol provides accurate quantitative (total mass of the contaminating MPs in the sediment sample, with separate quantification for each polymer type, as specified below) and qualitative (type of polymer) information with sensitivities orders of magnitude higher than those attainable with the general methodology most commonly adopted so far by researchers worldwide. The methods described in the literature, based on MPs separation from the sediment by flotation in a high density saline solution (NaCl, NaI, Na tungstate, etc.) followed by quantification and characterization typically by means of optical microscopy and micro-spectroscopy techniques [23], may suffer from inaccuracy due to the underestimation caused by the missed detection of MPs below the mesh size of the filtering device, and to the overestimation caused by residual biogenic and inorganic contaminating material. In particular, the PISA protocol allows quantification of the total mass of MPs, regardless of their size and morphology, that are constituted by the following polymers: polyolefins, PS, PET, and the two polyamides nylon 6 (polycaprolactame, the homopolymer of AHA) and nylon 6,6 (copolymer of HMDA, with adipic acid) [24]. These are also the main commodity polymers and, not incidentally, are also considered to be the main macro- and microplastic marine pollutants.

Although techniques similar to those comprised in the PISA protocol have been described, they do not include the complete set of relevant polymers; in particular, pressurized solvent extraction [25] is quite effective for polyolefins and other polymers soluble in common solvents (mainly PS and other vinyl polymers) but misses PET and polyamides that are the most abundant microplastics from synthetic textile fibers, while a previously described depolymerization of PET followed by high performance liquid chromatography (HPLC) coupled with mass spectrometry [26] does not include polyamides. On the other hand, a thermoanalytical method based on the accurate quantification of the total carbon content from synthetic polymers recently proposed by J. Lin et al. [27], besides losing the information on size and shape as in the PISA protocol, does not allow specification of the type of polymeric materials in the contaminating MPs and only provides an estimation of the total amount of microplastics due to the different fractional carbon content in each polymer type.

The benthic sediment samples analyzed in the present work were collected in two close locations of the sublittoral southern Ligurian Sea close to the harbor of Leghorn and the estuary of the Arno river, Italy, and in particular within the shoals of the Meloria protected marine area and in nearby shallow coastal waters, respectively. The sediments were sieved at 2 mm mesh and then submitted to a sequence of fractional solvent extractions with refluxing dichloromethane (DCM) and xylene (Xy) as selective solvents for PS and polyolefins, respectively [28], followed by sequential hydrolytic depolymerization of polyamides, under acidic conditions, and of PET, under alkaline conditions. Although the extracted polyolefins and PS are quantified by gravimetry and pyrolysis-GC/MS, the total content of nylon 6, nylon 6,6, and PET are calculated from the quantitative analysis, by reversed-phase HPLC, of their respective monomers: the two amines AHA and HMDA after tagging with a fluorophore [29], and terephthalic acid (TPA) [30]. Differently from the previously reported examples in which this procedure (or part of it) had been tested on less complex sandy beach sediment or less demanding (because of the high MPs content) wastewater treatment plant sludges, this is the first report on the PISA procedure for the detection and quantification of MPs in benthic sediments.

## 2. Materials and Methods

### 2.1. Sediment Sampling

Benthic (bottom) marine sediment samples were collected in four sites of relatively shallow waters of the continental shelf in the Ligurian Sea along the northern coastline of Tuscany, Italy (Table 1). The sampling was performed on 3 July 2018, using a single corer 10 cm in diameter. After collection, the top ~5 cm of the sediment was placed in glass flasks with metal lid and then air-dried in a laminar flow hood in the lab and stored in a fridge at 2 °C. The subsequent analyses were performed in second half of 2020. Care was taken as to avoid contamination from airborne and other environmental MPs; for this purpose, all glassware was rinsed with the given solvent (previously filtered on 0.45 μm pore size membrane) prior to use; all open surfaces of solutions and solid samples and extracts were kept covered with aluminum foils throughout the various manipulations except during the actual operations and transfer; personal protective equipment included cotton protective coats.

**Figure 1:**
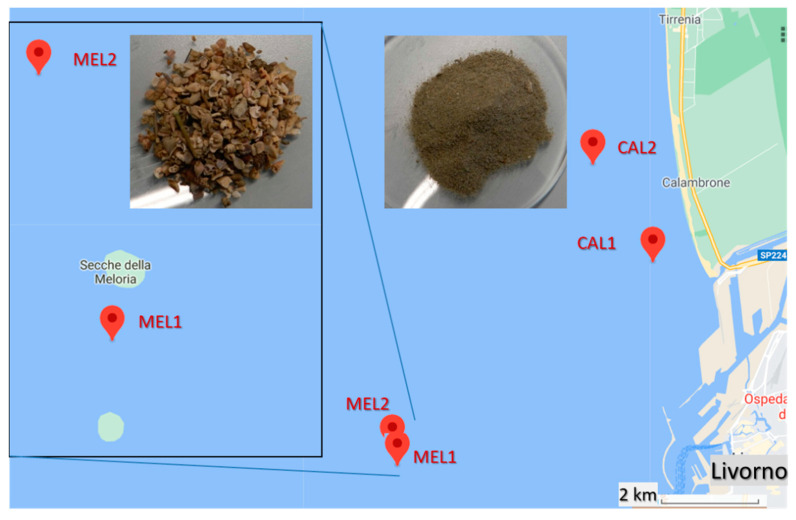
Sampling sites and morphology of the two types of sediment sample: CAL and MEL.

The sedimentologic features are representative of two distinct benthic zones: the MEL samples (as identified in Table 1) were collected in the shoals of the marine Protected Area “Secche della Meloria”, about 3 miles west of the harbor of Leghorn, with the bottom sediments consisting mainly of fragmented organogenic shells and carbonate sand in a seabed partially covered by meadows of *Posidonia oceanica*, a seagrass endemic to the Mediterranean Sea also known as Mediterranean tapeweed; the CAL samples were collected northeast of the MEL area, in the upper shore platform about 0.5–1 mile off of the sandy beaches of Calambrone, characterized by intensive seasonal touristic presence, and 5 miles south of the Arno river estuary, resulting in the bottom sediments consisting of very fine sandy to silty material (Figure 1). The sampling sites were chosen as they are influenced by the currents carrying the estuarine waters of the Arno river, the main river in the Tuscany region collecting wastewaters from about 2,200,000 inhabitants and from several industrial districts (tanning and textile, among others), and by the commercial and touristic harbor of Leghorn. Moreover, the benthic sediments of the Meloria shoals are quite peculiar as they are strongly affected by surface currents while being possibly too far from the coastline to collect high density debris carried by the riverine freshwaters.

### 2.2. Chemicals

Dichloromethane (DCM, 99.9%, stabilized with amylene, Romil-SpS, Romil Ltd., Cambridge, UK), xylene (Xy, 98.5%, Sigma-Aldrich, Merck Life Science S.r.l., Milano, Italy, hereafter Sigma-Aldrich), methanol (99.9%, Sigma-Aldrich), acetic acid (99.85% Sigma-Aldrich), sulfuric acid (95–98%, Sigma-Aldrich), hydrogen peroxide (30% *w/v*, Panreac, Nova Chimica Srl, Cinisello Balsamo, Italy), 6 N aqueous hydrochloric acid (prepared from 37% HCl, Sigma-Aldrich), 1.9 N aqueous sodium hydroxide (from NaOH pellets, 98.0%, Sigma-Aldrich), hexadecyl-tributyl-phosphonium bromide (TBHDPB, 97%, Sigma-Aldrich), HPLC-grade water (Sigma-Aldrich), and reversed-phase Solid Phase Extraction (SPE) cartridges (Chromabond^®^ C18ec loaded with 500 mg stationary phase, Macherey-Nagel GmbH & Co., Düren, Germany) were used for sediment extractions, extracts purifications, and for the acid and alkaline depolymerizations of the hydrolysates. Dansyl chloride (DNS-Cl, 96%, Alfa Aesar Thermo Fisher (Kandel) GmbH, Kandel, Germany), *n*-butyl amine (99.5%, Honeywell Fluka Chemicals, Fisher Scientific Italia, 20053 Rodano, Italy, hereafter Fluka) and potassium carbonate (K_2_CO_3_, Carlo Erba Reagents S.r.l., 20010 Cornaredo, Italy) were used in the dansylation of AHA and HMDA amino monomers. Chloroform (HPLC-grade stabilized with ethanol, Sigma-Aldrich) was used as mobile phase in size exclusion chromatography (SEC) analysis. Acetonitrile (HPLC-grade, ≥99.9%, Sigma-Aldrich), HPLC-grade water (Sigma-Aldrich), triethyl amine (≥99.9%, Fluka), acetic acid (99.85%, Sigma-Aldrich), methanol (99.9%, Sigma-Aldrich), and phosphoric acid (Sigma-Aldrich) were used in the preparation of the HPLC eluents for determination of dansylated amine monomers in the acid hydrolysates for the quantitative analysis of terephthalic acid in the alkaline hydrolysates.

### 2.3. Analytical Techniques

SEC analyses were performed with an instrument consisting of a Jasco (Jasco Europe Srl, Cremella, LC, Italy) PU-2089 Plus four-channel pump, a PLgel pre-column packed with polystyrene/divinylbenzene and two in series PLgel MIXED-D columns (Agilent Technologies Italia S.p.A., 20063 Cernusco sul Naviglio, Italy) placed in a Jasco CO_2063 column oven, a Jasco RI 2031 Plus refractive index detector, and a Jasco UV-2077 Plus multi-channel UV spectrometer. Chloroform was used as the eluent at 1 mL/min flow rate.

Pyrolysis-Gas Chromatography/Mass spectrometry (Py-GC/MS). Analyses were performed using a multi-shot pyrolyzer EGA/PY-3030D (Frontier Laboratories Ltd., Koriyama 963-8862, Japan) coupled with a 6890N gas chromatographic system with a split/splitless injection port and combined with a 5973-mass selective single quadrupole mass spectrometer (Agilent Technologies, Inc., Santa Clara, CA 95051, USA). For the analysis of the extracts, 100–150 µL of solution were placed in stainless steel cups together with 2.0 μL of a solution of dibutyl phthalate-3,4,5,6-d_4_ (500 ppm), and dried under nitrogen steam prior to the analyses [31]. The temperatures used for the double shot pyrolysis were 350 °C and 600 °C while the interface was set at 280 °C. The GC injection port temperature was 280 °C. The GC injection was operated in split mode with a split ratio of 1:10. For the analysis of particles, the pyrolysis was performed in a single shot at 600 °C with the interface set at 280 °C and split ratio 1:10 [32]. The chromatographic and mass spectrometric conditions were as follows: 5 min isotherm at 50 °C, heating up to 180 °C at 12 °C/min, 2 min isotherm, heating up to 300 °C at 8 °C/min, and 20 min isotherm; 1.2 mL/min He (99.9995%) carrier gas; GC/MS interface temperature 280 °C and MS electron ionization voltage 70 eV. Perfluorotributylamine (PFTBA) was used for mass spectrometer tuning. MSD ChemStation D.02.00.275 software (Agilent Technologies, Inc., Santa Clara, CA 95051, USA) was used for data analysis and the peak assignment was based on a comparison with libraries of mass spectra (NIST 8.0).

Infrared spectra in the mid-IR region (700–4000 cm^−1^) were recorded with a Perkin Elmer Spectrum Autoimage System microscope equipped with an Attenuated Total Reflectance (ATR) module with germanium crystal; each spectrum is the result of 64 scans accumulation at 4 cm^−1^ spectral resolution. The lateral spatial resolution corresponds to the contact area with the germanium crystal tip (30–40 micron).

Two instrumental setups were used for HPLC. Quantitative determination of TPA was performed using a Jasco PU-1580 isocratic pump connected with a Jones-Genesis Aq 120 reversed-phase column (150 mm × 4.6 mm, 4 μm particle size) operating at room temperature and a Jasco 1575 UV-Vis detector (UVD) set at 242 nm wavelength. Analyses were carried out on 20 μL of the solutions at 0.8 mL/min flow rate of an isocratic 30/70 vol/vol methanol/HPLC-grade water (acidulated with 0.1 wt.% phosphoric acid) eluent. The DNS-Cl derivatives of AHA and HMDA were analyzed with an Agilent 1260 Infinity Binary LC instrument equipped with pre-column, a reversed-phase Phenomenex-Aqua C18 column (250 mm × 4.6 mm, 5 μm particle size) and diode array (DAD VL + 1260/G1315C, set at 335 nm wavelength) plus fluorescence (FLD 1260/G1321B, set at 335/522 nm excitation/emission wavelengths) double detector. Elution was performed at 1.0 mL/min flow rate in gradient mode by combining an aqueous solution of 2.5% acetic acid and 0.83% triethylamine (phase A) with acetonitrile (phase B) according to the program reported in Table 2.

### 2.4. Synthetyc Polymer Recovery by Sequential Extractions with Selective Solvents

Each sediment sample was submitted to a first extraction with refluxing dichloromethane (DCM), targeting the solubilization of polystyrene, followed by a second step in refluxing xylene to solubilize semicrystalline polyolefins. For this purpose, approximately 100 g of sediment that had been previously sieved at 2 mm and air-dried to nearly constant weight in equilibrium with the atmospheric lab conditions, was loaded into a cellulose thimble that was placed into a kumagawa apparatus and the extraction was performed for 2 h with 250 mL refluxing DCM. Before each extraction, the apparatus was conditioned by refluxing 100 mL DCM for 3 h to remove any contaminant. The DCM extract was reduced to 1–2 mL in a rotatory evaporator, then transferred into a 5 mL glass vial conditioned to constant weight in an oven at 60 °C and weighed. The residue from the DCM extraction was then further extracted in the same apparatus with 250 mL refluxing xylene. The obtained xylene solution was transferred into a two-necked flask fitted with distillation head and condenser, reduced to 5 mL by distilling off the excess solvent, and then added with 20 mL of 1.6 M KOH in methanol to induce the precipitation of polyolefins; the obtained precipitate, mainly consisting of PP, LDPE, HDPE, was then collected by vacuum filtration through a 0.22 μm Durapore PVDF membrane (without PP backing).

### 2.5. Hydrolytic Depolymerization Procedures

The total content of nylon 6, nylon 6,6, and PET contaminating microparticles (mainly found as microfibers) in the sediment samples was calculated from the content of the corresponding monomers 6-aminocaproic acid (AHA), 1,6-hexanediamine (or hexamethylenediamine, HMDA), and terephthalic acid (TPA), respectively, in the hydrolysates obtained from the acid and alkaline depolymerizations for the polyamides and the polyester, respectively. As nylons and PET are neither soluble in DCM nor in xylene, the solid residue from the organic solvent extraction was analyzed for their quantification. In particular, the dried residue of the organic solvent extraction of each sample was transferred into a 100 mL round-bottomed flask equipped with a reflux condenser and magnetic stirring bar. The selective determination of nylons and PET was based on the sequential selective hydrolysis of all aliphatic polyamides in acidic conditions, and of PET in alkaline conditions.

**Figure 2:**
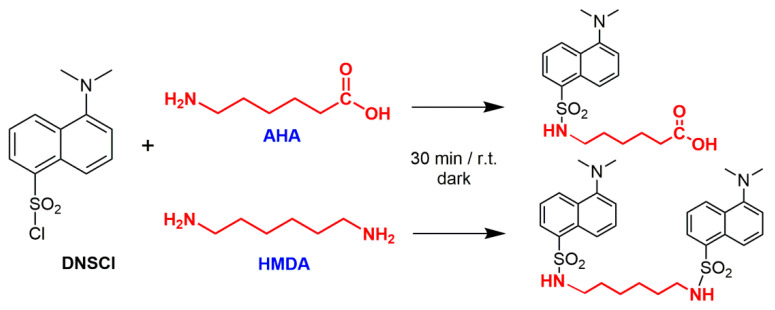
Dansylation of the amino-monomers AHA and HMDA from the depolymerization of nylon 6 and nylon 6,6.

After addition of approximately 80 mL 6 N HCl the stirred mixture was heated to the reflux temperature of about at 105 °C for 24 h. At the end of the hydrolysis, the reaction mixture was vacuum-filtered on a 0.22 μm PVDF membrane to separate the solid residue from the acid solution. The filter membrane with the solid residue was carefully rinsed with small amounts of HPLC-grade water for the subsequent treatments, while the acid solution was transferred in a 100 mL volumetric flask and taken to volume with 6 N HCl. A given volume (5 mL) of the obtained solution was weighed and neutralized to pH 6.5–7.5 with 5 N NaOH. To enable a highly sensitive quantification of the amino-monomers AHA and HMDA, the solutions were treated with 5-dimethylaminonaphtalene-1-sulfonyl chloride (dansyl chloride, DNS-Cl), a derivatizing fluorophore commonly used in protein sequencing (Figure 2).

For the derivatization of AHA and HMDA, 1 mL of the neutralized product of acid hydrolysis was loaded in a 5 mL glass vial, added with 1.0 mL aqueous K_2_CO_3_ solution (80 g/L), to favor the precipitation of calcium carbonate if present in the neutralized solution. After allowing the obtained mixture to settle, 1 mL was taken and added with a further 1.0 mL aqueous K_2_CO_3_ solution and 1.0 mL of a 5 g/L solution of DNS-Cl in acetone (18.5 μmol). After 30 min stirring at room temperature in the dark, an excess of *n*-butyl amine (5.0 μL, 51 μmol) was added to quantitatively convert the unreacted DNS-Cl. The solution containing the derivatized amines (including those from the hydrolysis of both natural and synthetic polyamides) was then transferred into a 10 mL volumetric flask and taken to volume with a 1:1 (*v*/*v*) water/acetone mixture before HPLC analysis.

For the determination of the PET content, the solid residues collected at the end of the acid hydrolysis were treated under alkaline hydrolytic conditions to achieve the complete PET depolymerization. For this purpose, each residue was rinsed with de-ionized water on the same PVDF membrane used for filtration, then transferred into a 100 mL round-bottomed flask equipped with a reflux condenser and magnetic stirring bar, added with 40 mL 1.9 N NaOH and TBHDPB as a phase transfer catalyst, then the mixture was stirred at 85 °C for 6 h. The final solution was vacuum-filtered on a 0.22 μm PVDF membrane, then transferred into 50 mL volumetric flasks and taken to volume with 1.9 N NaOH. For the removal of most of the residual biogenic contaminants that might interfere with the subsequent purification by elution through a SPE cartridge (e.g., by saturating the adsorption capacity of the cartridge), 1 mL of hydrolysate was weighed at 0.1 mg accuracy, transferred into a 10 mL glass vial with 1–2 mL of 30 vol% H_2_O_2_ until complete discoloration and/or end of visible bubble formation, then added with 1 mL 1.9 M H_2_SO_4_. The resulting acidic solution was further purified to remove potential interferents before HPLC analysis; for this purpose, the pre-treated hydrolysate was eluted through a reversed-phase SPE cartridge, the adsorbate was then desorbed with 0.8 mL MeOH and the recovered roughly 0.8 mL solution in methanol was weighed at 0.1 mg accuracy. Finally, 0.5 mL of the solution was taken up with a micropipette, placed in a vial and weighed again at 0.1 mg accuracy, then added with 0.75 mL aqueous CH_3_COOH (1 wt.% in HPLC-grade water) to obtain a 40/60 vol% methanol/water mixture.

**Figure 3:**
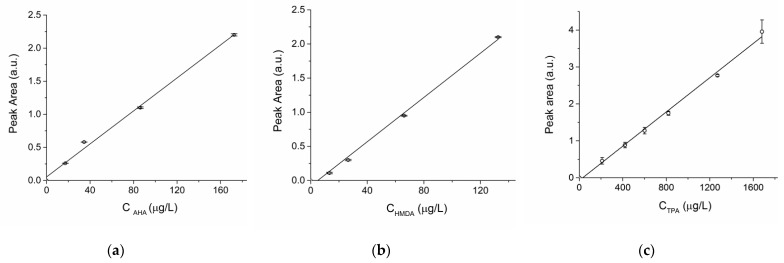
Linear regressions of the calibration dataset for the quantitative determination of the monomeric units: (**a**) AHA, with FLD (fitting parameters: peak area = 0.01246 × C_AHA_ + 0.05411; R2 = 0.99424); (**b**) HMDA, with FLD (fitting parameters: peak area = 0.01622 × C − 0.08077; R2 = 0.99466); (**c**) TPA, with UV detector (fitting parameters: peak area = 231955.9 × C − 7236.6; R2 = 0.99532). Each calibration was performed by running the measurements in triplicate.

**Figure 4:**
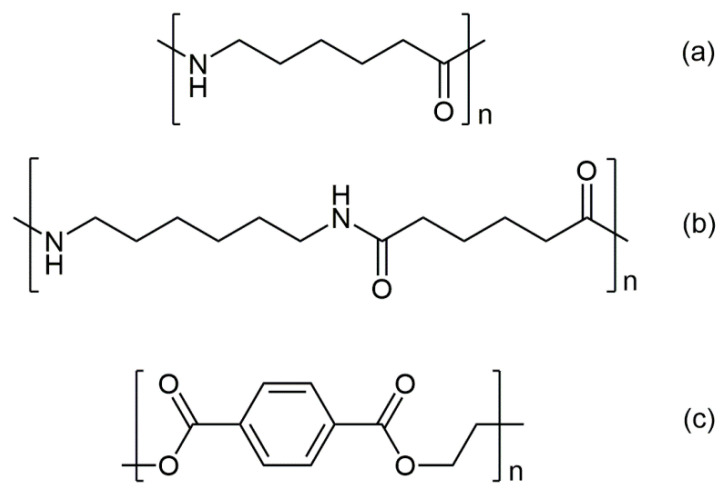
Polymer repeating units: (**a**) nylon 6 (polycaprolactame, the homopolymer of AHA); (**b**) nylon 6,6 (copolymer of adipic acid and HMDA); (**c**) PET (polyethylene terephthalate).

The amounts of nylon 6, nylon 6,6 and PET in each sample (given in ppm, or mg polymer/kg dry sludge) was calculated from the corresponding monomer concentration C_AHA_, C_HMDA_, and C TPA (in ppm) as determined by HPLC, based on the calibrated response of both UV and fluorescence detectors (see Figure 3), according to Equations (1)–(3):

(1) $$\mathrm{nylon}\;6\;(\mathrm{ppm})\;=\;C_{\mathrm{AHA}}\cdot \frac{MW_{PA6}}{MW_{AHA}}$$

(2) $$\mathrm{nylon}\;6,6\;(\mathrm{ppm})\;=\;C_{\mathrm{HMDA}}\cdot \frac{MW_{PA6,6}}{MW_{HMDA}}$$

(3) $$\mathrm{PET}\;(\mathrm{ppm})\;=\;C_{\mathrm{TPA}}\cdot \frac{MW_{PET}}{MW_{TPA}}$$

where *MW_PA_*_6_ = 113.16 g/mol, *MW_PA_*_6,6_ = 226.32 g/mol, and *MW_PET_* = 192.2 g/mol, are the molecular weights of the repeating units in the corresponding polymer (Figure 4), and *MW_AH_*_A_ = 131.17 g/mol, *MW_HMDA_* = 116.21 g/mol, and *MW_TPA_* = 166.13 g/mol those of the analytes.

### 2.6. Calibrations

Calibration of the response of the pyrolysis-gas chromatography-mass spectrometry (Py-GC/MS) system used for PS quantification in the DCM extract was performed by analyzing a set of PS solutions prepared starting from a 100 ppm solution of PS in DCM, then weighed amounts of calibration solution with a total PS content in the 20−150 μg range were loaded in the crucible and dried. The quantification was based on the response (GC/MS peak) for the fragment corresponding to the styrene dimer [32], which gave a linear regression of the experimental calibration (r^2^ = 0.9998), from which the following limit of detection (LOD) and limit of quantification (LOQ) were calculated: LOD = 3·*SD*/*m* = 0.10 µg; LOQ = 10·*SD*/*m* = 0.35 µg (with *SD* standard deviation of the blank areas, and *m* slope of the calibration curve).

For the HPLC analyses of the dansylated amines the FLD detector response was calibrated against the concentration of dansylated AHA by recording a 4-point calibration using solutions in the 17.25–172.5 µg/L range plus a blank sample, all in triplicate; the same procedure was followed for dansylated HMDA, in the 13.25–132.5 µg/L range plus the blank sample. From the linear regression (Figure 3a,b) obtained for dansylated AHA (A = 1.246 · 10^−2^ · C_AHA_ + 5.4 · 10^−2^; r^2^ = 0.99784) and dansylated HMDA (A = 1.682 · 10^−2^ · C_HMDA_ − 8.195 · 10^−2^; r^2^ = 0.99791) the following values were calculated: LOD_AHA_ = 0.903 μg/L; LOQ_AHA_: 3.910 μg/L; LOD_HMDA_ = 0.301 μg/L; LOQ_HMDA_: 0.758 μg/L. The LOD and LOQ values are given as:

(4) $$LOD=\frac{standard\;deviation\;of\;most\;diluited\;solution\;}{slope\;of\;linear\;regression\;}\cdot 3;$$

(5) $$LOQ=\frac{standard\;deviation\;of\;most\;diluited\;solution\;}{slope\;of\;linear\;regression\;}\cdot 10.$$

For the HPLC quantification of TPA a linear calibration of the UV detector response was obtained by recording a 6-point calibration based on standard TPA solutions in 2N NaOH in the 0.21–1.68 mg/L range plus a blank, all in triplicate [23]. From the linear regression (Figure 3c, A = 2.32·10^5^·C_TPA_ − 7237; r^2^ > 0.995) the following values were calculated: LOD = 0.117 mg/L; LOQ: 0.391 mg/L. (calculated in this case as the ratio between the concentration, or the calibrated peak area, and the signal-to-noise ratio, times 3 for LOD and times 10 for LOQ; the blank sample gave no detectable peak).

## 3. Results

The overall procedure for the determination of the total mass of individual polymer types that are present as microparticles and fragments in the sediment involves a preliminary step of sieving to recover the fraction below 2 mm in size and air drying, followed by a first sequence of extractions with boiling solvents that are selective for the hydrocarbon polymers. In particular, extraction with DCM (boiling point b.p. = 39.6 °C) allows recovery of the amorphous polystyrene, along with most low-molecular-weight (MW) organic compounds (both biogenic, such as fats, and synthetic, such as plasticizers and surfactants) and the oligomeric fraction deriving from the extensive photo- and thermal oxidation of polyolefins (PE, PP, and olefin copolymers). In addition, most vinyl polymers such as e.g., acrylics, polyvinyl chloride (PVC) and polyvinyl acetate may be co-extracted in boiling DCM, but their presence as microplastic contaminants in marine sediments is likely to be negligible because they are not (or no longer in the case of PVC) commonly used in the disposable items and packaging materials that are by far the main contributors to the plastic waste reaching the marine environment. The second extraction of the residue from the DCM extraction is performed in boiling xylene (b.p. = 139 °C), to recover the less oxidized and high-MW polyolefins, possibly along with some proteins that may be co-extracted. The extractable fraction may then be further purified to remove the biogenic fraction, and analyzed by one or more techniques for the quantification of the total mass of each polymer type.

The subsequent steps, consisting of a sequence of hydrolytic treatments performed under acid and then alkaline conditions, are performed under optimized conditions to selectively and sequentially achieve the complete depolymerization of all aliphatic polyamides (both synthetic and natural) and all polyesters, respectively. The resulting hydrolysates may then be submitted to further purification (different environmental matrices may require different purification procedures) before performing reversed-phase HPLC analysis that allows the accurate and sensitive quantification of the monomers and to calculate the corresponding amount of the original polymer. In the case of the polyamides, an additional tagging of the amino-monomers with a fluorophore is performed prior to the HPLC analysis to increase of orders of magnitude the sensitivity of the measurement. The overall procedure had been previously validated on different matrices (marine beach and underwater lakebed sediments [28,30], wastewater treatment plant sludges [29]) by performing microplastic spiking and recovery experiments.

### 3.1. MPs Fractionation by Polymer Type through Selective Solvent Extraction

#### 3.1.1. Polystyrene and Highly Degraded Hydrocarbon Polymers

The DCM extracts are expected to contain not only PS, but also highly oxidized and degraded polyolefin oligomers and other vinyl polymers less frequently found as microplastic pollutants (e.g., polyacrylates, PVC, etc.), in addition to biogenic low-MW species. The total amounts of DCM extractable fraction in the four samples are reported in Table 3. Further extractions with refluxing xylene to collect the DCM-insoluble, less degraded polyolefin fraction gave in most cases very small amounts of dry matter that could be neither weighed with sufficient accuracy nor further purified, and were therefore not further analyzed; the only exception was the xylene extract from CAL2, from which a sizable solid particulate could be recovered (see Section 3.1.2).

**Figure 5:**
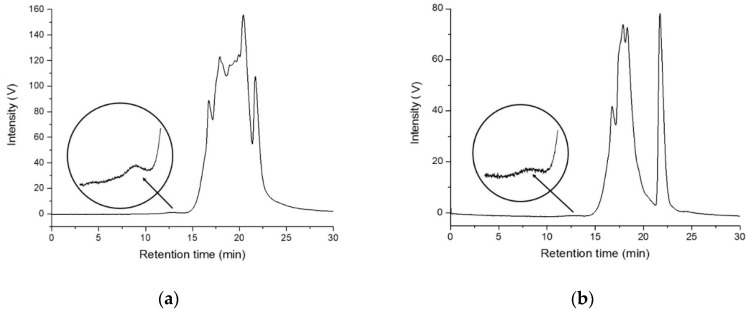
Representative examples of the SEC traces (the curves displayed are those recorded with UV detector set at 260 nm) for the DCM extractable fractions of: (**a**) MEL1; (**b**) MEL2.

The dried DCM extracts were picked up with chloroform to about 5 mg/mL and the obtained solutions were microfiltered (to remove any contamination by inorganic particles) and analyzed by SEC. The chromatographic profiles obtained with UV detectors set at 260 nm and 340 nm always show the presence of a main very broad and structured peak at retention times r.t. > 15 min due to low-MW polymers and oligomers along with other low MW species; an additional weak peak roughly centered at r.t. ≈ 12.5 min could be detected for samples MEL1, MEL2, and CAL1, corresponding to higher MW polymers (Figure 5 and Table 4).

The SEC-UV detector response for the high MW fractions in MEL1, MEL2, and CAL1 is characterized by a strong absorption at 260 nm that becomes negligible at 340 nm, as one would expect from polystyrene [33], but differently from most nonaromatic polymers.

**Figure 6:**
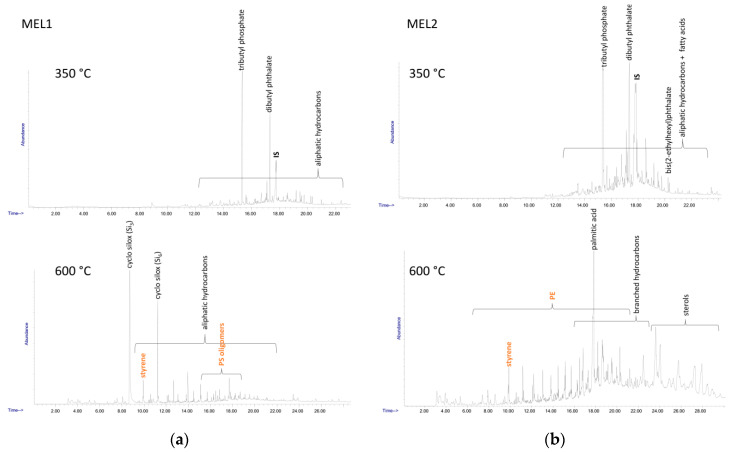
Py-GC-MS chromatograms of sediment DCM extracts: (**a**) MEL1; (**b**) MEL2.

**Figure 7:**
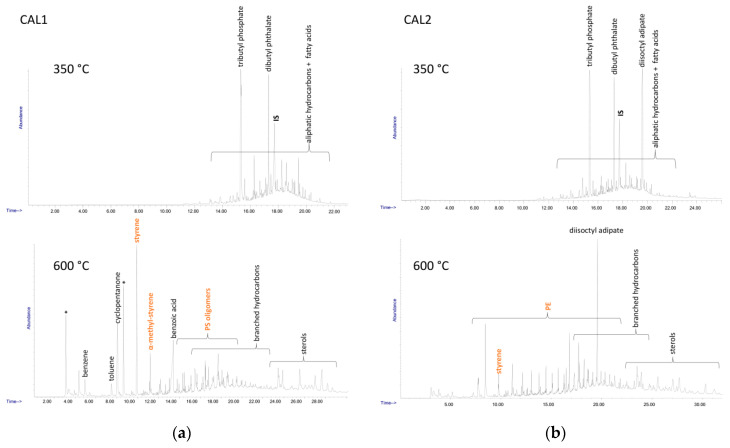
Py-GC-MS chromatograms of sediment DCM extracts: (**a**) CAL1; (**b**) CAL2.

The overall PS content in the sediment samples (including the low MW oligomeric fraction) was determined by double shot-Py-GC/MS analysis performed on the DCM extracts, according to a calibration based on the MS count corresponding to the PS dimer fragment; the results are reported in Table 3. The double shot technique also allows separate detection of different species; in the first shot at lower temperature (here 350 °C) low MW compounds such as hydrocarbon species deriving from highly degraded polyolefins or plasticizers and other common plastics additives are typically observed, along with some polystyrene oligomers, while the presence of other synthetic polymers could be detected in the second shot (here 600 °C). The main species identified in the DCM extracts of the four samples are listed in Table 5, while the Py-GC/MS chromatograms recorded after each shot are shown in Figure 6 and Figure 7.

The Py-GC/MS chromatogram obtained at 350 °C from the MEL1 extract was mainly characterized by the presence of tributyl phosphate (TBP) and dibutyl phthalate (DBP), two nearly ubiquitous environmental pollutants largely used as plasticizers in many applications. The pyrolysis products from the high temperature shot were mainly the typical markers of PS (styrene and its low oligomers) and of polysiloxane.

Similarly, in all the other MEL and CAL extracts the 350 °C shot resulted in the release of various plasticizers such as TBP, DBP, bis(2-ethylhexyl) phthalate (BEHP) and diisooctyl adipate (DOA), along with naturally occurring fatty acids. At 600 °C the pyrolysis markers of PS were always detected, along with those of PE (only in the case of the extracts from MEL2 and CAL2); finally, various sterols of likely natural origin, and branched hydrocarbons possibly originating from the degradation of synthetic surfactants could also be detected.

#### 3.1.2. High Molecular Weight Polyethylenes (HDPE, LDPE) and Polypropylene

**Figure 8:**
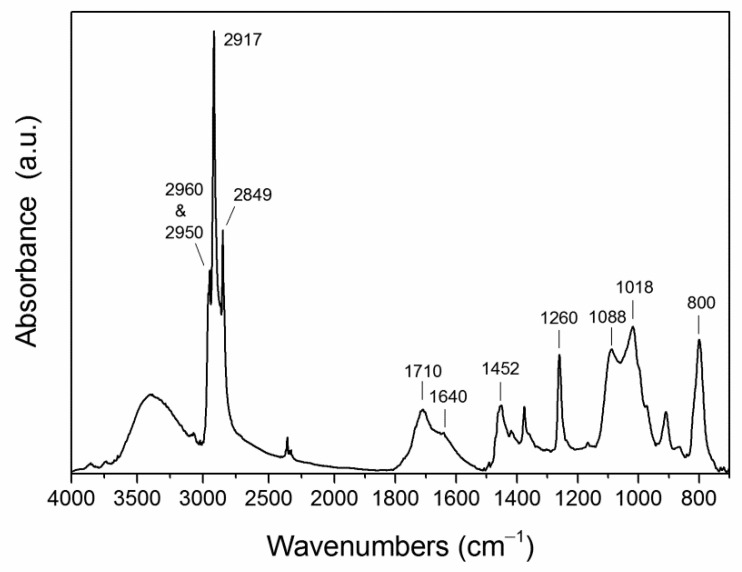
Micro-ATR-FTIR spectrum of the xylene-extractable fraction of CAL2.

For the semi-quantitative determination of semi-crystalline polyolefin MPs (polyethylenes and polypropylene) that are insoluble in DCM unless highly oxidized and degraded to low molecular weights, the residues from DCM extraction were further extracted with refluxing xylene. After distilling off most of the xylene from the final extracts they were added with an excess of a solution of KOH in methanol and the precipitate was then collected by filtration, dried, and weighed. A quantifiable number of precipitated particles (2.9 mg from 81.6 g of sediment) was only recovered from sample CAL2. The micro-ATR FTIR spectrum in Figure 8 clearly indicates that the precipitate mainly consists of oxidized polyethylene (methylene CH asymmetric and symmetric stretching bands at 2917 and 2849 cm^−1^, respectively; weak methyl CH stretchings at 2960 and 2866 cm^−1^, methylene bendings at 1452, and 1375 cm^−1^), with a high oxidation level shown by the intense and broad carbonyl absorption with main peaks at 1710 and 1660 cm^−1^ (isolated and conjugated aldehydes and ketones generated by photo-oxidation and subsequent chain cleavage reactions) and the broad absorption centered at 3400 cm^−1^ from hydroxyl groups. Further absorptions can be ascribed at least partially to polydimethylsiloxane (methyl deformation at 1260 cm^−1^, symmetric and asymmetric Si–O–Si stretchings at 1088 and 1018 cm^−1^, and Si–C stretching at 800 cm^−1^, in addition to a small C–H stretching peak at 2950 cm^−1^) possibly due to contamination by silicone grease during the lab operations.

### 3.2. Total Mass Content of Polyamide (nylon 6 and nylon 6,6) and Polyester (PET) Mps by Depolymerization and Quantitative Analysis of the Resulting Comonomers

For the quantification of nylon 6 and nylon 6,6 polyamides the solid residues from the sequential extractions with DCM and Xy were treated with refluxing 6 N HCl to achieve the total depolymerization of both natural and synthetic polyamides. Due to the high carbonate content of the two MEL sediments samples a high volume of HCl had to be slowly added to allow complete evolution of CO_2_ upon conversion of the carbonate mineral into the corresponding chlorides. The acid hydrolysate solutions were separated from the residue by filtration on 0.22 µm PVDF membranes, neutralized and treated with the fluorescent tag dansyl chloride (DNS-Cl) before reversed-phase HPLC analysis, as described in detail in Section 2. The solid residues from the acid hydrolysis were then treated with 1.9 N NaOH to achieve quantitative depolymerization of PET MPs, followed by purification of the alkaline hydrolysate and quantification of the TPA content by reversed-phase HPLC analysis, as described in detail in Section 2.

Table 6 reports the detected concentrations of the dansylated AHA and HMDA and of TPA from which the concentration of nylon 6, and nylon 6,6, and PET MPs in the sediment samples (the air-dried sediments were considered to be a starting material) could be calculated.

### 3.3. Analysis of Microplastic’s Particles Detected on Filter Membrane

**Figure 9:**
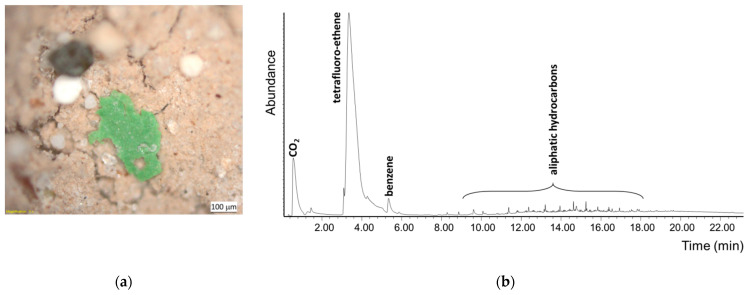
PTFE microparticles found in the inorganic sediment residue after all extraction and hydrolysis proceduresperformed on the MEL1 sample: (**a**) micrograph of a green microparticle taken with a stereomicroscope; (**b**) pyrogram of the microparticle.

The final residue recovered from the filter in last step of the overall procedure was observed under an optical microscope to detect the presence of any microplastic particle resistant to all the extraction and hydrolysis processes. In the case of the MEL1 sample, a few sub-millimeter sized green plastic fragments weighing about 50 µg each could easily be detected in the brown-greyish inorganic residue (Figure 9). The fragments were identified as polytetrafluoroethylene (PTFE) from the presence of a tetrafluoroethene main peak in the Py-GC/MS chromatogram recorded from each individual particle.

## 4. Discussion

The complete PISA protocol for the separation, purification, and quantification of the total mass contents of PS, polyolefins, PET, nylon 6 and nylon 6,6 MPs in environmental matrices, recently developed and previously applied in the analysis of the contamination level in sandy shore sediments and in wastewater treatment plant sludges, has been successfully applied for the first time to benthic marine sediments, considered to be the final sink of most of the plastic waste inflow in the oceans.

The selection of the polymer types to be investigated, which dictated the design of the overall separation, fractionation, and analysis scheme, was based on the considerations, supported by an increasing number of scientific papers and technical reports, that the most abundant polymer types in benthic marine sediments correspond to those that are also produced globally in larger amounts. These are: polyolefins and polystyrene, largely used in short lifetime applications such as packaging and single use disposable items, and therefore likely to end up as unmanaged plastic waste; the two main synthetic polymer classes used as staple textile fibers or as materials of fishery and aquaculture activities, i.e., is the polyester PET and the two polyamides nylon 6 and nylon 6,6, included among the target polymers because textile fibers released in laundering wastewaters and mismanaged fishing gears are well recognized threats for the marine ecosystems.

**Figure 10:**
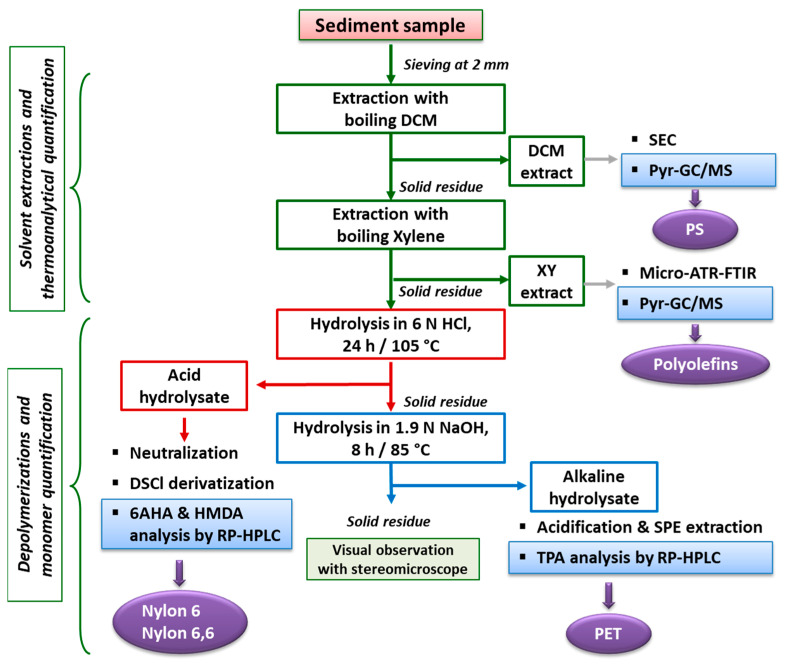
Flowchart of the entire analytical protocol for the separate quantification of the total mass of micro- and nanoparticles of different polymer types.

The overall procedure, schematically shown in the flowchart of Figure 10, allows the tackling of the two main challenges faced when such polymeric materials end up in sea bottom sediments, either because of their high density or as a result of photo-oxidation and/or biofouling promoting vertical transport down the water column. These are the lengthy (and possibly inaccurate) procedures for the density separation of the MPs from the sediment, and the size threshold for their detection by micro-spectroscopy techniques, a possibly critical issue in particular for the low-density polyolefins and PS. Indeed, the latter hydrocarbon polymers are likely to reach the benthic sediments only once they have undergone significant degradation, which may include fragmentation down to the sub-micrometer size range, well below the detection limit of a few micrometers and up to tens of micrometers typical of the micro-spectroscopy techniques commonly used for MPs in sediments.

Among the most noteworthy results presented here are the successful implementation of an improved version of the procedure for the quantification of PS, and the results confirming the presence of both low-MW and high-MW low-density polyolefins in benthic sediments. In particular, the Pyr-GC/MS technique adopted for the quantification of PS allows improvement to improve the accuracy with respect to the previously reported procedure based on FD-SEC [33], in which only the high MW fraction could be evaluated due to the interference by biogenic species and oxidized polyolefin molecular fragments in the low molecular weight fraction. The presence of PS in the 8–65 ppm concentration range could thus be determined with accuracy, while the MW distribution determined by SEC analysis highlighted the presence of a significant fraction of degraded low-MW PS, in agreement with the expected presence in the bottom sediment of low-density hydrocarbon polymers that have undergone significant photo-oxidation. The presence of highly oxidized high-MW polyolefin MPs, although in very small amount, clearly indicates that polymer oxidation contributes to their vertical transport and ultimate deposition due to their increased density and hydrophilicity.

The results obtained in this work also highlight the versatility of the procedure, which deal with sediments of very different compositions (silicate silt and coarse organogenic carbonate debris).

Finally, the double shot Py-GC/MS technique allowed separate detection in the DCM extracts of all samples various phthalates and other low-MW plasticizers (suspect endocrine disruptors), while the high-MW fraction was found to contain, in addition to PS, also polysiloxane (in the DCM extract of MEL1) possibly from silicones used in formulations of personal care products, and degraded fractions of polyethylene (in MEL2 and CAL2). Although not mentioned before, the likely presence of biodegradable (not necessarily so once in the marine sediment) aliphatic-aromatic polyesters could also be identified from the presence of some markers of poly(butylene terephthalate-co-adipate).

The hydrolytic depolymerization of the higher density heteropolymers (PET, nylon 6 and nylon 6,6) followed by HPLC analysis of the resulting monomers allowed accurate quantification of the contamination level presumably associated with the deposition of synthetic textile fibers carried by urban wastewaters. The somewhat surprising higher level of contamination by polyamides (2.7–12.1 ppm) compared to PET (1.5–0.2 ppm) may be the result of different sources of pollution (e.g., fishing gears), although the number of analyzed samples was too small to allow drawing general and accurate considerations. In fact, while PET was detected in all the analyzed samples, polyamides were only detected in some of them. Finally, the presence of PTFE particles isolated from the final residue could be the result of a point source or of a specialized source of pollution, as this material is widely used in technical fishing equipment.

Although the methodology used in this work cannot provide information on important parameters such as the number, size, and shape of the individual plastic particles, it provides important complementary and unique quantitative information allowing the gaining of an accurate picture on the transport, extent, and distribution of MPs in the marine environment, thus clarifying the actual role of the sea bottom as MPs sink.

## Figures and Tables

**Table 1:** Sample acronyms and relevant sampling sites coordinates and depth.

Sample	Acronym	Geolocalization		Depth (m)
Meloria 1	MEL1	43°32′50.0″ N 10°13′08.2″ E	43.547219 lat. 10.218944 lon.	3
Meloria 2	MEL2	43°33′1,02″ N 10°13′4,03″ E	43.5502778 lat. 10.2177778 lon.	4
Calambrone 1	CAL1	43°35′5,21″ N 10°17′2,34″ E	43.5847778 lat. 10.2839722 lon.	20
Tirrenia-Calambrone 2	CAL2	43°36′9,67″ N 10°16′7,80″ E	43.6026944 lat. 10.2688333 lon.	17

**Table 2:** Elution program adopted for the analysis of dansylated amine derivatives.

Elution Time (min)	Mobile Phase (A) %	Mobile Phase (B) %	Elution Mode
0 → 20	60	40	isocratic
20 → 25	60 → 30	40 → 70	gradient
25 → 35	30	70	isocratic
35 → 37	30 → 60	70 → 40	gradient
37 → 50	60	40	isocratic

**Table 3:** Extractable fraction in DCM from the sediment samples.

Sediment Sample	Extracted Sediment (g)	Extractables (mg)	Total Extract ^1^ (ppm)	PS Content ^2^ (ppm)
MEL1	82.85	6.2	75	8
MEL2	107.96	12.8	119	11
CAL1	100.91	9.1	90	65
CAL2	112.57	10.7	95	16

^1^ Total concentration expressed as mg of dry extractable matter per kg dry sediment. ^2^ Determined by double shot-Py-GC/MS measurements, from the styrene dimer peak and the corresponding instrumental calibration.

**Table 4:** SEC analysis of the DCM extractable fractions in the sediment samples.

Sample	Retention Time ^1^ (min)	${\bar{M}}_{n}\;\;(g\cdot {\mathrm{mol}}^{-1})$	${\bar{M}}_{w}\;\;(g\cdot {\mathrm{mol}}^{-1})$	PDI ^2^
MEL1	12.83	35,883	42,245	1.18
	20.41	92	367	4.0
MEL2	12.22	36,855	48,336	1.31
	17.89	254	585	2.30
CAL1	12.71	29,653	46,250	1.56
	17.88	270	529	1.96
CAL2	n.d. ^3^	n.a. ^3^	n.a. ^3^	n.a. ^3^
	18.04	196	475	2.43

^1^ peak value reported here only as an aid for better visualization; ^2^ Polydispersity Index PDI = ${\bar{M}}_{w}/{\bar{M}}_{n}$; ^3^ n.d.= not detectable (below the limit of detection, LOD); n.a. = not applicable.

**Table 5:** Most abundant species identified by double shot-Py-GC/MS in the DCM extracts.

Acronym	First Shot (350 °C) ^1^	Second Shot (600 °C)
MEL1	TBP, DBP	PS, siloxane
MEL2	TBP, DBP, BEHP, fatty acids	PS, PE, branched hydrocarbons, sterols
CAL1	TBP, DBP, fatty acids	PS, sterols, branched hydrocarbons
CAL2	TBP, DBP, DOA	PS, PE, sterols, branched hydrocarbons

^1^ TBP = tributyl phosphate; DBP = dibutyl phthalate; BEHP = bis(2-ethylhexyl) phthalate; DOA = diisooctyl adipate.

**Table 6:** Concentration of PA’s monomers and relative polymers in sediment sample.

Acronym	AHA ^1^ (μg/L)	Nylon 6 ^2^ (ppm)	HMDA ^1^ (μg/L)	Nylon 6,6 ^2^ (ppm)	TPA ^1^ (mg/L) ^1^	PET (ppm) ^2^
MEL1	n.d. ^3^	n.a. ^3^	n.d.	n.a.	0.101	290
MEL2	n.d.	n.a.	n.d.	n.a.	0.0489	137
CAL1	36.6	11.2	8.97	2.7	0.633	1523
CAL2	35.0	12.1	n.d.	n.a.	0.061	174

^1^ Concentration of the monomer (or its dansyl derivative in the case of the two amines) in the solution obtained after purification of the corresponding acid (for the two amines) or alkaline (for TPA) hydrolysate. ^2^ Total concentration in ppm (mg polymer/kg dry sediment) as calculated from the detected amount of the corresponding monomers. ^3^ n.d.= not detectable (below the limit of detection, LOD); n.a. = not applicable.
